# “Hot cross bun” is a potential imaging marker for the severity of cerebellar ataxia in MSA-C

**DOI:** 10.1038/s41531-021-00159-w

**Published:** 2021-02-15

**Authors:** Shuzhen Zhu, Bin Deng, Zifeng Huang, Zihan Chang, Hualin Li, Hui Liu, Yanjun Huang, Ying Pan, Yanping Wang, Yin-Xia Chao, Ling-Ling Chan, Yih-Ru Wu, Eng-King Tan, Qing Wang

**Affiliations:** 1 Department of Neurology, Shunde Hospital of Southern Medical University, Foshan, Guangdong P.R. China; 2 Department of Neurology, Zhujiang Hospital of Southern Medical University, Guangzhou, Guangdong P.R. China; 3 Department of Neurology, the Second Affiliated Hospital of Guangzhou Medical University, Guangdong, China; 4 Department of Neurology, National Neuroscience Institute, Singapore General Hospital, Duke-NUS Medical School, Singapore, Singapore; 5 Department of Neurology, Chang Gung Memorial Hospital, Linkuo Medical Center and Chang Gung University College of Medicine, Taoyuan, Taiwan; 6 Guangdong-Hong Kong-Macao Greater Bay Area Center for Brain Science and Brain-Inspired Intelligence, Guangdong, China

**Keywords:** Parkinson's disease, Neurodegenerative diseases

## Abstract

To evaluate the correlation between “hot cross bun” sign (HCBs) and disease severity in multiple system atrophy (MSA). We recruited patients with probable and possible MSA with parkinsonism (MSA-P) or the cerebellar ataxia (MSA-C) subtypes. Clinical and imaging characteristics were collected and comparison was performed between MSA-C and MSA-P cases. Spearman test was used to evaluate the correlation between HCBs and other variables. Curve estimate and general linear regression was performed to evaluate the relationship between HCBs and the Scale for Assessment and Rating of Ataxia (SARA). Unified Multiple System Atrophy Rating Scale (UMSARS) IV was used to assess the severity of disease. Multinomial ordered logistic regression was used to confirm the increased likelihood of disability for the disease. Eighty-one MSA with HCBs comprising of 50 MSA-C and 31 MSA-P were recruited. We demonstrated that the severity of HCBs showed a positive linear correlation with SARA scores in MSA-C. Multinomial ordered logistic regression test revealed that the increase in the HCBs grade may be associated with an increased likelihood of disability for the disease severity in MSA, especially in those with cerebellar ataxia subtype. We demonstrated that HCBs is a potential imaging marker for the severity of cerebellar ataxia. The increase in the HCBs grade may be associated with an increased likelihood of disability in MSA-C, but not MSA-P cases, suggesting that it may be a useful imaging indicator for disease progression in Chinese patients with MSA-C.

## Introduction

Multiple system atrophy (MSA), is an adult-onset progressive age-related neurodegenerative disease, characterized by the presence of a 14 kDa protein α-synuclein inclusion. The clinical characteristics and neuro-pathogenesis of MSA are similar to those in Parkinson’s Disease (PD)^1–3^. There are two clinical forms including the parkinsonism subtype (MSA-P) with predominant parkinsonism, and cerebellar subtype (MSA-C) with predominant cerebellar ataxia in early stages. The pathology in MSA is mainly found in striato-nigral, olivopontocerebellar, and spinal cord structures and its pathological hallmark is glial cytoplasmic inclusions^4,5^. Conventional magnetic resonance imaging is frequently used in the diagnosis of MSA, and “hot cross bun” sign (HCBs) is widely accepted to be a valuable sign for the diagnosis of MSA^6–14^. HCBs signal appears to be more pronounced as the disease progresses, suggesting that it may be a useful imaging indicator for disease progression. However, there is limited information on the exact relationship between HCBs signal and clinical phenotypes in MSA. Carré et al.^15^ in a recent study did not find a correlation between magnetic resonance imaging (MRI) features and the Scale for Assessment and Rating of Ataxia (SARA) in MSA patients. However, the study subjects were Caucasians and the predominant subtype is MSA-P in the study, and there were only 11 MSA-C patients.

To address the gap in current literatures, we investigated HCBs-positive MSA patients among mainland ethnic Han Chinese and profiled the clinical phenotypes in HCBs-positive MSA patients, assessed the relationship between HCBs and the severity of ataxia and independent viability. In addition, we explored whether there was heterogeneity in neuroimaging and clinical correlation between two subtypes (MSA-P vs MSA-C).

## Results

### Demographic characteristics and clinical phenotypes in HCBs-positive MSA patients

Eighty-one patients including 50 (62%) MSA-C and 31 (38%) MSA-P were included (Table 1). Among MSA-C patients, 32 cases (64%) were probable MSA and 18 cases (36%) were possible MSA. Among MSA-P patients, 16 cases (51.7%) were probable MSA and 15 cases (48.3%) were possible MSA. Gender distribution and duration of diseases were not significantly different between patients with MSA-P and those with MSA-C (Table 1). Age at symptom onset and enrollment showed a significant difference between patients with MSA-P and those with MSA-C (Table 1). As expected, parkinsonian manifestation was more prevalent in cases with MSA-P and cerebellar symptoms were more prevalent in those with MSA-C, and autonomic failure was present in all cases (Table 2). The ratio of dementia in our MSA cases is ~15% (Table 2), which is similar to the report by O’Sullivan et al. 2008^14^. The severity of cerebellar ataxia and disability differed significantly between patients with MSA-P and MSA-C (Table 2). The score of SARA and UMSARS-IV was higher in participants of MSA-C than those in MSA-P (Table 2). The Fleiss’ kappa and Cohen’s Kappa for inter and intra-rater reliability is 0.750 and 0.815. The Inter and intra-rater reliability for the grade of HCBs was showed in Supplementary Table 1. Neither HCBs grade nor atrophy of pons and MCP differed between MSA-C and MSA-P (Table 2).

**Table 1:** Demographic characteristics of MSA patients.

	Overall	MSA-C	MSA-P	*p* value
	(*n* = 81)	(*n* = 50)	(*n* = 31)	
*Sex*				0.293^†^
Woman	30 (38%)	22 (44%)	10 (32%)	
Men	51 (62%)	28 (56%)	21 (68%)	
*Age*				
Enrollment	57.5 (7.1)	56.0 (6.6)	59.8 (7.4)	0.018^‡a^
Symptoms onset	55.7 (7.2)	53.5 (6.4)	58.5 (7.5)	0.005^‡^^a^
Duration of disease	2.0 (1.0–3.0)	2.0 (1.0–2.3)	2.0 (1.0–4.0)	0.370^§^

Comparison was conducted between MSA-C and MSA-P groups. Data are *n* (%), mean SD, or median (IQR), unless stated otherwise.

^†^Based on *χ*^2^ test, ^‡^Based on *t* test, and ^§^Based on Mann–Whitney test with significance level of 0.05 (two-tailed).

^a^Significant difference between MSA-C and MSA-P patients, *P* < 0.05.

*MSA-C* multiple system atrophy (cerebellar subtype), *MSA-P* multiple system atrophy (parkinsonism subtype).

**Table 2:** Clinical and imaging characteristics were compared between MSA-C and MSA-P patients with HCBs.

	Overall (*n* = 81)	MSA-C (*n* = 50)	MSA-P (*n* = 31)	*p* value
*Autonomic failure*				
Orthostatic hypotension	44 (54%)	30 (60%)	14 (45%)	0.193^†^
Urinary incontinence	8 (10%)	4 (8%)	4 (13%)	0.474^‡^
Incomplete bladder empty	49 (60%)	28 (56%)	21 (68%)	0.293^†^
Constipation	53 (65%)	33 (66%)	20 (65%)	0.891^†^
Any	81 (100%)	50 (100%)	31(100%)	….
*Cerebellar dysfunction*				
Gait ataxia	68 (84%)	47 (94%)	21 (68%)	0.004^‡^^a^
Limb ataxia	68 (84%)	46 (92%)	22 (71%)	0.026^‡^^a^
Cerebellar dysarthria	28 (35%)	23 (46%)	5 (16%)	0.008^‡^^a^
Nystagmus	8 (10%)	8 (16%)	0 (0%)	0.021^‡^^a^
Any	74 (91%)	50 (100%)	24 (77%)	0.001^‡^^a^
*Parkinsonism*				
Bradykinesia	43 (53%)	14 (28%)	29 (94%)	0.001^‡^^a^
Rigidity	39 (48%)	12 (24%)	27 (8%)	0.001^‡^^a^
Postural instability	46 (57%)	25 (50%)	21 (68%)	0.117^†^
Resting tremor	37 (46%)	10 (20%)	27 (87%)	0.001^‡^^a^
Any	48 (59%)	17 (34%)	31(100%)	0.001^‡^^a^
*Corticospinal dysfunction*				
Babinski sign	43 (53%)	25 (50%)	18 (58%)	0.480^†^
Atypical symptoms				
Dementia	12 (15%)	8 (16%)	4 (12%)	0.760^‡^
*Neurological scores*				
SARA	11.0 (4.0–16.5)	14.0 (5.8–21)	5.0 (2.0–13.0)	0.001^§^^a^
UMSARS-IV	2.0 (2.0–4.0)	3.5 (2.0–4.0)	3.0 (2.0–5.0)	0.775^§^
*Imaging features*				
HCB vertical line	1.57 (1.44–1.71)	1.58 (1.45–1.71)	1.56 (1.35–1.70)	0.434^§^
HCB horizon line	1.49 (0–1.89)	1.73 (0–1.99)	0.95 (0–1.70)	0.169^§^
Atrophy of pons	1.66 (1.48–1.89)	1.67 (1.48–1.87)	1.66 (1.48–1.89)	0.694^§^
Atrophy of MCP	1.05 (0.89–1.24)	1.04 (0.92–1.23)	1.08 (0.85–1.34)	0.953^§^
HCB grade	3 (2–4)	3 (2–4)	3 (2–4)	0.465^§^

Data are *n* (%), or median (IQR), unless stated otherwise.

^†^Based on *χ*^2^ test, significance level of 0.05 (two-tailed). ^‡^Based on Fisher’s exact test (if any cell has fewer than ten observations) with level of significance set to 0·05 (two-tailed). ^§^Based on Mann–Whitney test with significance level of 0.05.

^a^Significant difference between MSA-C and MSA-P patients, *P* < 0.05.

*UMSARS* Unified Multiple System Atrophy Rating Scale, *MSA-C* multiple system atrophy (cerebellar subtype), *MSA-P* multiple system atrophy (parkinsonism subtype). *MCP* middle cerebellar peduncles. *SARA* Scale for Assessment and Rating of Ataxia.

### HCBs positively correlated with the severity of ataxia in MSA -C patients

Spearman’s correlation coefficient for ranked data analysis was performed in the MSA, MSA-C, and MSA-P cases. The correlation analysis between the HCBs grade and disease duration was non-significant in MSA patients (rho = 0.179, *p* = 0.093). However, the HCBs grade was significantly correlated with cerebellar ataxia severity (SARA) (rho = 0.594, *p* < 0.001) global disability (UMSARS-IV) (rho = 0.560, *p* < 0.001). It is worth noting that a stronger correlation was found between the HCBs and SARA (rho = 0.784, *p* < 0.001) as well as UMSARS-IV (rho = 0.794, *p* < 0.001) in MSA-C patients than in MSA patients. However, the HCBs was not significantly associated with the SARA or UMSARS-IV in MSA-P patients. The detailed data are shown in Table 3.

**Table 3:** Correlation analysis of HCBs grade with variables in MSA patients.

Variables	Overall	(*n* = 81)	MSA-C	(*n* = 50)	MSA-P	(*n* = 31)
	*r*	*p*^†^	*r*	*p*^†^	*r*	*p*^†^
*Autonomic failure*						
Orthostatic hypotension	−0.085	0.453	−0.061	0.675	−0.179	0.335
Urinary incontinence	0.119	0.288	0.139	0.337	0.089	0.636
Incomplete bladder empty	0.043	0.706	−0.026	0.859	0.195	0.294
Constipation	0.180	0.108	0.177	0.220	0.186	0.316
*Cerebellar dysfunction*						
Gait ataxia	0.177	0.114	0.021	0.885	0.314	0.086
Limb ataxia	0.308	0.005	0.183	0.203	0.442	0.013
Cerebellar dysarthria	0.261	0.019	0.319	0.024	0.091	0.627
Nystagmus	−0.135	0.230	−0.194	0.178	…	…
*Parkinsonism*						
Bradykinesia	−0.041	0.719	0.084	0.563	−0.219	0.236
Rigidity	−0.116	0.304	−0.043	0.766	−0.183	0.325
Postural instability	−0.161	0.152	−0.163	0.258	−0.123	0.509
Resting tremor	−0.059	0.604	0.110	0.447	−0.277	0.132
*Corticospinal dysfunction*						
Babinski sign	0.174	0.120	0.196	0.173	0.166	0.373
*Atypical symptoms*						
Dementia	−0.081	0.471	−0.110	0.446	−0.022	0.906
*Neurological Scores*						
SARA	0.594	<0.001	0.788	<0.001	0.326	0.073
UMSARS-IV	0.560	<0.001	0.794	<0.001	0.154	0.408
*Imaging features*						
Atrophy of pons	−0.311	0.005	−0.271	0.057	−0.357	0.048
Atrophy of MCP	−0.411	<0.001	−0.383	0.006	−0.434	0.015
Disease duration	0.179	0.093	0.109	0.520	0.376	0.037
Age on symptom onset	−0.061	0.587	−0.582	0.573	−0.132	0.479

^†^Based on Spearman test, significance level of 0.05 (two-tailed).

*UMSARS* Unified Multiple System Atrophy Rating Scale, *MSA-C* multiple system atrophy (cerebellar subtype), *MSA-P* multiple system atrophy (parkinsonism subtype), *MCP* middle cerebellar peduncles.

**Fig. 1:**
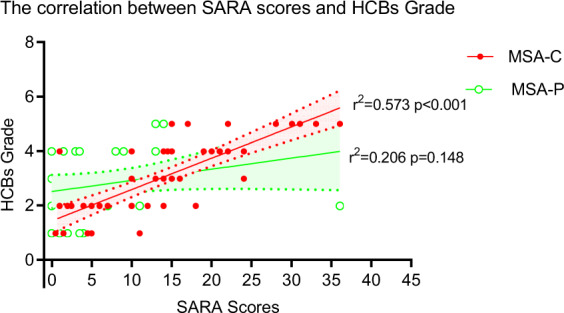
The Scatter plot showed the correlation between SARA sum score and HCBs grade. Each dot represented on case. The red solid dot represented case in MSA-C group and the green hollow dot represented case in MSA-P group. 95% confidential interval (CI) was shown in shadow area.

**Fig. 2:**
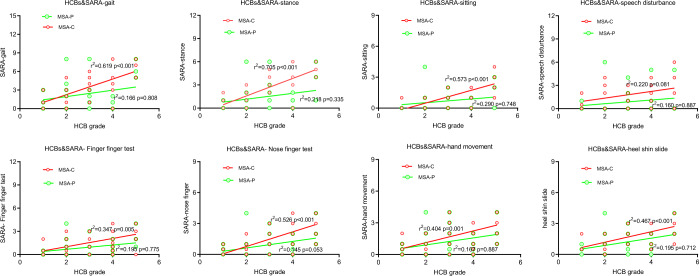
Relationship between HCBs and the severity of ataxia in MSA patients. The Scatter plot showed the correlations between scores of each item of SARA and HCBs grade. Each dot represented on case. The red dot represented cases in MSA-C group and the green dot represented cases in MSA-P group.

The correlation analysis between the HCBs grade and atrophy of pons, middle cerebellar peduncles (MCP) were also assessed in our study. The HCBs grade was significantly correlated with atrophy of pons (rho = −0.311, *p* = 0.0005) and MCP (rho = −0.411, *p* < 0.001) in MSA patients. The significant correlations between HCBs and MCP was also found in MSA-C and MSA-P patients (Table 3). A General Linear Model with univariate analysis showed that there was a significant positive linear correlation between the HCBs grade and SARA scores (adjusted *R*^2^ = 0.573, *P* < 0.001) in MSA-C cases after adjusted for atrophy of pons, atrophy of MCP and disease duration (Table 4, Fig. 1 and Fig. 2).

**Table 4:** Relationship between HCBs and severity of ataxia as assessed by score of sums and each item of SARA.

Variables	Overall	(*n* = 81)	*p*^†^	MSA-C	(*n* = 50)	*p*^†^	MSA-P	(*n* = 31)	*p*^†^
	*r*^2^	Adjusted *r*^2^		*r*^2^	Adjusted *r*^2^		*r*^2^	Adjusted *r*^2^	
SARA	0.451	0.339	<0.001	0.634	0.573	<0.001	0.392	0.206	0.148
Gait	0.510	0.432	<0.001	0.705	0.619	<0.001	0.444	0.166	0.808
Stance	0.511	0.449	<0.001	0.759	0.705	<0.001	0.485	0.298	0.335
Sitting	0.462	0.410	<0.001	0.634	0.573	<0.001	0.394	0.290	0.748
Speech disturbance	0.304	0.205	0.221	0.363	0.220	0.081	0.389	0.167	0.887
Finger–finger test	0.386	0.308	0.005	0.467	0.347	0.005	0.409	0.195	0.775
Nose–finger test	0.454	0.393	<0.001	0.603	0.526	<0.001	0.373	0.145	0.953
Fast alternating hand movements	0.383	0.314	0.002	0.502	0.404	0.001	0.389	0.167	0.887
Heel–shin slide	0.397	0.330	0.001	0.554	0.467	<0.001	0.410	0.195	0.772

^†^Based on General Linear Model with univariate analysis, atrophy of pons, atrophy of MCP and disease duration were analyzed as covariates. significance level of 0.05 (two-tailed).

*UMSARS* Unified Multiple System Atrophy Rating Scale, *MSA-C* multiple system atrophy (cerebellar subtype), *MSA-P* multiple system atrophy (parkinsonism subtype).

### An increase in the HCBs grade is associated with an increased likelihood of disability in MSA-C patients

Recent studies suggested that many factors, such as age of onset^16–18^, gender^17,18^, disease duration^17^, and atrophy of pons^17^, might involve in the progression of MSA; however, owing to the differences of population and main end-point indicators in studies, the conclusions of these studies were inconsistent. In our study, age of onset, gender, disease duration, and atrophy of pons were included for analysis.

To investigate these factors, multinomial ordered logistic regression test was performed. Possible risk factors, such as age at symptom onset, sex, disease duration, HCBs, atrophy of pons, atrophy of MCP were included into the analysis. We showed that the increase in the HCBs grade may be associated with an increased likelihood of disability in MSA-C patients after adjusting for sex, age at symptom onset, atrophy of pons, atrophy of MCP and disease duration (Table 5). With the increase of HCBs grade, the risk of severe disability gradually increased (Table 5). The risk of disability in MSA-C patients with HCBs grade 5 was 35.71, 83.33, and 500 times higher than that those with grade 4, 3, and 2 (Table 5). Age of onset, disease duration, and atrophy of pons were not associated with an increased likelihood of disability in MSA patients in our study (Table 5).

**Table 5:** Factors associated with disabilities in MSA patients.

Risk factors	OR	Overall	(*n* = 81)	OR	MSA-C	(*n* = 50)	OR	MSA-P	(*n* = 31)
		*p*^†^	OR 95% CI		*p*^†^	OR 95% CI		*p*^†^	OR 95% CI
*Age*									
Symptom onset	1.020	0.551	0.955–1.089	0.937	0.206	0.846–1.037	1.095	0.141	0.970–1.237
*Sex*									
Woman	3.889	0.008	1.428–10.591	3.058	0.121	0.745–12.5	10.7531	0.013	1.667–71.429
Man	1								
Disease duration	1.002	0.986	0.802–1.252	1.003	0.988	0.694–1.448	0.920	0.652	0.639–1.323
*Atrophy*									
pons	0.184	0.229	0.012–2.898	0.068	0.267	0.001–7.826	0.445	0.691	0.008–24.074
MCP	7.232	0.159	0.460–113.683	2.865	0.658	0.027–304.440	7.284	0.337	0.127–418.949
*HCB grade*									
1	0.005	<0.001	0.001–0.041	0.000	<0.001	0.000–0.005	0.016	0.031	0–0.686
2	0.016	<0.001	0.002–0.100	0.002	<0.001	0.000–0.026	0.121	0.232	0.004–3.864
3	0.019	<0.001	0.003–0.116	0.012	0.001	0.001–0.164	0.010	0.008	0–0.295
4	0.068	0.002	0.013–0.371	0.028	0.004	0.003–0.311	0.088	0.132	0.004–2.080
5	1			1			1		

^†^Based on Multinomial Ordinal logistic regression test, significance level of 0.05 (two-tailed). *MSA-C* multiple system atrophy (cerebellar subtype), *MSA-P* multiple system atrophy (parkinsonism subtype), *MCP* middle cerebellar peduncles, *OR* odds ratio.

## Discussion

There is limited literature on the correlation of HCBs with clinical manifestations and disease severity. Our study identified several novel observations. We showed that in MSA-C, the HCBs grade was positively correlated with the severity of cerebellar ataxia as assessed by SARA. The increase in the HCBs grade may be associated with an increased likelihood of disability in MSA-C patients as assessed by UMSARS-IV. In our large cohort, we also highlighted that HCBs may be a useful imaging indicator only for MSA-C subtype progression and severity but not for the parkinsonism subtype. MSA-C subtype appears more prevalent in Asians than in Caucasians^19^.

Some investigators suggested that the microstructural changes within the transverse and longitudinal fibers predated the formation of the HCBs and may be correlated with the MSA clinical presentations^20^. Here we analyzed the correlation between the HCBs grade and the severity of cerebellar ataxia. Spearman’s correlation showed that the HCBs grade was significantly correlated with each SARA item in MSA-C patients, including gait, stance, sitting, speech disturbance, finger chase, and nose–finger tests (Table 4 and Fig. 2). Linear regression analysis showed that the HCBs grade and SARA scores had a positive linear relationship. These findings demonstrated that the high signal on T2-weighted images of HCBs could be a potential indicator of the severity of cerebellar ataxia in MSA-C subtype.

A “HCB” sign in the pontine basis (PB) was seen in 63.3% MSA patients. These pontine abnormalities became more prominent as MSA-C features advanced. Watanabe, et al.^18^ found that the atrophy of PB showed a significant correlation particularly with the interval following the appearance of cerebellar symptoms in MSA-C (*r* = 0.76 and *P* < 0.01), but the relationship between atrophy and functional status was highly variable among the individuals. In this study, we further evaluated the relationship between the atrophy of pons and middle cerebellar peduncles with functional status (disability) and no relationship was found. Owing to the fact that the occurrence of HCBs was earlier than the obvious atrophy of pons and could be graded according to the disease severity, indicating HCBs may be a more sensitive imaging indicator. In our study, among the case with grade 1–3, 28.6% was severe disability; however, among the case with grade 4–5, the proportion jumped to 78.1%. ROC curve analysis showed the AUC is 0.76 (0.65–0.86).

One recent study in Caucasians reported no correlation between MRI features and SARA in MSA-C^15^. Methodological differences may explain the varying result of our study. The study included MSA-C cases with and without HCBs signatures; however, we only included MSA-C cases with confirmed HCBs signatures. Furthermore, there were only 11 patients with MSA-C subtype compared with 51 cases in our study.

Interestingly, we did not find any correlation between the HCBs and SARA scores in the MSA-P subtype, strongly suggesting differences in pathophysiology between C and P subtypes. Based on the clinical manifestations of MSA-C and MSA-P in previous reports and our study, these two subtypes have great clinical heterogeneity^21^. Although the grade of HCBs was not different between MSA-C and MSA-P in our cohort, we only observed the significant but mild correlation between limb ataxia and HCBs grade (Table 3) in MSA-P cases. However, in all other variables of MSA-P cases, no significant correlations between HCBs grade and variables were found, suggesting that HCBs may not be a good indicator for the severity in MSA-P subtype. These findings suggested that these two subtypes might share different underlined pathological mechanisms; however, the exact cause of this heterogeneity deserved further study. We investigated the factors in MSA, and found that the increase in the HCBs grade may be associated with an increased likelihood of disability in MSA-C patients after adjusting other variables (Table 5).

Our study has some inherent limitations. The hospital-based setting of our study may have resulted in a selection bias and hence likely to underestimate the general prevalence of HCBs. We evaluated the HCBs grade in one-time point and did not measure the progression of cases. Although we select the superior cerebellar peduncle level of pons for measurement to keep the consistency between different individuals, the length of the horizontal line and vertical line of HCBs may vary depending on the level (height) of the axial image to be evaluated. Our study has certain strengths. We demonstrate that HCBs grade was positively correlated with the severity of cerebellar ataxia but not in those with parkinsonism. We also demonstrated that HCBs may be an indicator for disability in MSA-C patients after adjustment for age and atrophy of pons.

In conclusion, we demonstrated that HCBs grade was positively correlated with the severity of cerebellar ataxia in MSA-C and the increase in the HCBs grade may be associated with an increased likelihood of disability in MSA-C cases, strongly suggesting that HCBs grade may be a useful imaging indicator for the MSA-C subtype progression and severity. In MSA-P cases, HCBs was not an indicator for the disease severity, indicating that the underlying pathological mechanisms between MSA-C and MSA-P patients are different.

## Methods

### Study design and participants

We recruited patients from the Chinese Parkinson’s disease alliance, Department of Neurology of Zhujiang hospital, Southern Medical University. Images of MSA patients with HCBs on T2-weighted MRI were prospectively collected over a 7-year period. MSA cases were diagnosed according to the clinical criteria described by Gilman et al.^22^, and Low et al.^23^. Criteria for the diagnosis of probable MSA is that a sporadic, progressive, adult (>30 y)–onset disease characterized by autonomic failure involving urinary incontinence (inability to control the release of urine from the bladder, with erectile dysfunction in males) or an orthostatic decrease of blood pressure within 3 min of standing by at least 30 mm Hg systolic or 15 mm Hg diastolic and poorly levodopa-responsive parkinsonism (bradykinesia with rigidity, tremor, or postural instability) or a cerebellar syndrome (gait ataxia with cerebellar dysarthria, limb ataxia, or cerebellar oculomotor dysfunction). Criteria for possible MSA is that a sporadic, progressive, adult (>30 y)–onset disease characterized by parkinsonism (bradykinesia with rigidity, tremor, or postural instability) or a cerebellar syndrome (gait ataxia with cerebellar dysarthria, limb ataxia, or cerebellar oculomotor dysfunction) and at least one feature, suggesting autonomic dysfunction (otherwise unexplained urinary urgency, frequency or incomplete bladder emptying, erectile dysfunction in males, or significant orthostatic blood pressure decline that does not meet the level required in probable MSA) and at least one of the additional features as follows: (1) Babinski sign with hyperreflexia or stridor for possible MSA-P or MSA-C; 2) rapidly progressive parkinsonism or poor response to levodopa or postural instability within 3 y of motor onset or gait ataxia, cerebellar dysarthria, limb ataxia, cerebellar oculomotor dysfunction or dysphagia within 5 y of motor onset or atrophy on MRI of putamen, middle cerebellar peduncle, pons, cerebellum or hypometabolism on FDG-PET in putamen, brainstem, cerebellum for possible MSA-P; (3) Parkinsonism (bradykinesia and rigidity) or atrophy on MRI of putamen, middle cerebellar peduncle, or pons or hypometabolism on FDG-PET in putamen or presynaptic nigrostriatal dopaminergic denervation on SPECT or PET for possible MSA-C. Patients with both MSA-C and MSA-P were designated by onset of first symptom (cerebellar ataxia or parkinsonism). Briefly, inclusion criteria were: (i) MSA-P or MSA-C patients; (ii) HCBs on T2-weighted MRI; (iii) age of symptom onset was >30 years old.

Our exclusion criterion was (i) cerebellar ataxia or parkinsonism or autonomic failure might be owing to other central nervous system disease, such as stroke or paraneoplastic syndrome or hereditary cerebellar ataxia; (ii) family history of ataxia or parkinsonism; (iii) classic pill-rolling rest tremor; (iv) clinically significant neuropathy; (v) hallucinations not induced by drugs; (vi) onset after age 75 year; (vii) white matter lesions suggesting multiple sclerosis. In our study, dementia was observed as a possible symptom of MSA patients and was not considered as a non-supporting feature for MSA patients. We conducted this according to (i) O’Sullivan, S. S. et al.^18^, and Iva Stankovic et al., Movement Disorders Society MSA (MODIMSA) Study Group^24^. At first, 86 cases were enrolled, but five cases were excluded (one case complicating with cerebellar peduncle infarction, two cases with family history, two cases showing positive paraneoplastic antibody). The study was approved by the ethics committees of the Zhujiang Hospital of Southern Medical University, China (2018-SJNK-002). Formal consent for publication were obtained from all participants. All participants have provided written informed consent to take part in the study.

### Clinical assessment

Clinical data including demographics (sex, age on enrollment and symptom onset, disease duration), and presence of dysautonomia [orthostatic hypotension (a reduction of systolic blood pressure by at least 30 mm Hg, or a reduction of diastolic blood pressure by at least 15 mm Hg after 3 min of standing from a previous 3-minute interval in the recumbent position), urinary incontinence, incomplete bladder empty (measurement of urine residual volume (RV) by ultrasound revealing incomplete bladder emptying of 100 ml as described by Gilman et al. and Low et al.), constipation, cerebellar dysfunction (gait ataxia, limb ataxia, cerebellar dysarthria, nystagmus), Parkinsonism (Bradykinesia, rigidity, postural instability, resting tremor), corticospinal dysfunction (Babinski sign), dementia (defined as impairment of at least two cognitive domains and leading to substantial and apparently permanent impairment of ability to perform tasks of daily living. (DSM-IV, 1994)^18,25^, neurological scores (SARA score, Unified Multiple System Atrophy Rating Scale (UMSARS-IV)). The severity of cerebellar ataxia was assessed using the SARA as described by Subramony^26^. Eight items are included in this scale, including gait, stance, sitting, speech disturbance, finger chase, nose–finger test, fast alternating hand movements, and heel–shin slide. The maximum score is 40, indicating the most serious ataxia. Global disability was assessed with the fourth part of the Unified Multiple System Atrophy Rating Scale IV (UMSARS-IV) in MSA patients as described by Wenning GK^27^. UMSARS-IV is a disability scale, which was graded as follows according to the severity of disability: 1. life is completely independent, and patients can finish all housework; 2. life is not completely independent, and some housework cannot be finished independently; 3. life is somewhat dependent, and more than half of housework cannot be finished independently; 4. life is very dependent on others, and only a small amount of housework can be finished independently; 5. self-care is impossible, and the patient is bedridden. Grade 1–3 was defined to mild disability, and Grade 4–5 defined as severe disability.

### Image analysis

**Fig. 3:**
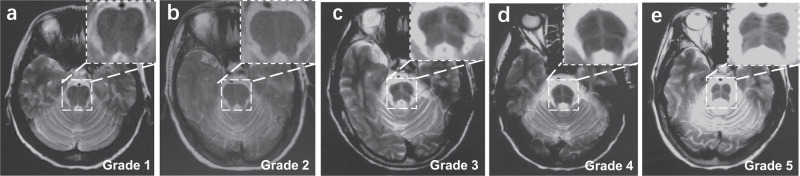
Representative imaging of MSA patients with HCBs. **a**–**e** Pontine “cross sign” stages 1–5. **a** Stage 1 at an emerging vertical T2 high signal line. **b** Stage 2 where the clear vertical line appeared. **c** Stage 3 where the horizontal line emerged. **d** Stage 4 where the horizontal line appeared as well as the vertical line completed (“cross” line completed). **e** Stage 5 where clear cross bun sign accompanied with pontine base atrophy.

Brain MRI examination was performed using a 3-T MR scanner (Achieva, Siemens Healthineers, Germany) with a 16-channel head coil. Three‐dimensional T1‐weighted images were acquired with axial orientation using a 3.0 T Discovery. Scan parameters were as follows: field of view (FOV): 230 × 184 × 96 mm; voxel: 1.64 × 1.64 × mm; Matrix: 140 × 112 × 48 slices; repetition time 3.7 ms, echo time of 1.69 ms, and slice thickness of 2 mm. The sequence type and acquisition parameters for the T2-weighted images were as follows: T2-weighted spin echo sequence: coil selection: SENSE-NV-16; FOV: AP 215 mm, RL 197 mm, FH 125 mm; Voxel size AP 0.575 mm, RL 0.72 mm, Slice thickness 7 mm, Stacks fold-over direction RL, TR 3000 ms, TE 80 ms. The grade of the HCBs was assessed according to criteria reported by Horimoto et al.^20^. The HCBs was graded as follows: no change appeared = 0; a vertical T2 high-signal line began to appear = 1; only a clear vertical line appeared = 2; a horizontal line began to appear following a vertical line appearance = 3; both a clear horizontal and a vertical line appeared (cross line completed) = 4; and the ventral pons in front of the horizontal line showed T2 high signal or the ventral pons decreased in size with pontine base atrophy = 5. Grade 1–3 was defined to unclear HCBs and 4–5 was defined to clear completed HCBs. The HCBs was graded by three independent neuroradiologists. For the intra-rater reliability study, before the evaluations, evaluators underwent the same 24-h training to standardize the evaluation procedure. After the training, a total of 30 participants were randomly selected. Every neuroradiologist evaluated HCBs twice and the intra-rater reliability was assessed. For the inter-reliability study, all participants were included. The three evaluators did not have access to the values of each other’s measurements. If the rating results are inconsistent, the final score can only be obtained after the agreement of at least two neuroradiologists. The representative images of different grade of HCBs were shown in Fig. 3a–e.

**Fig. 4:**
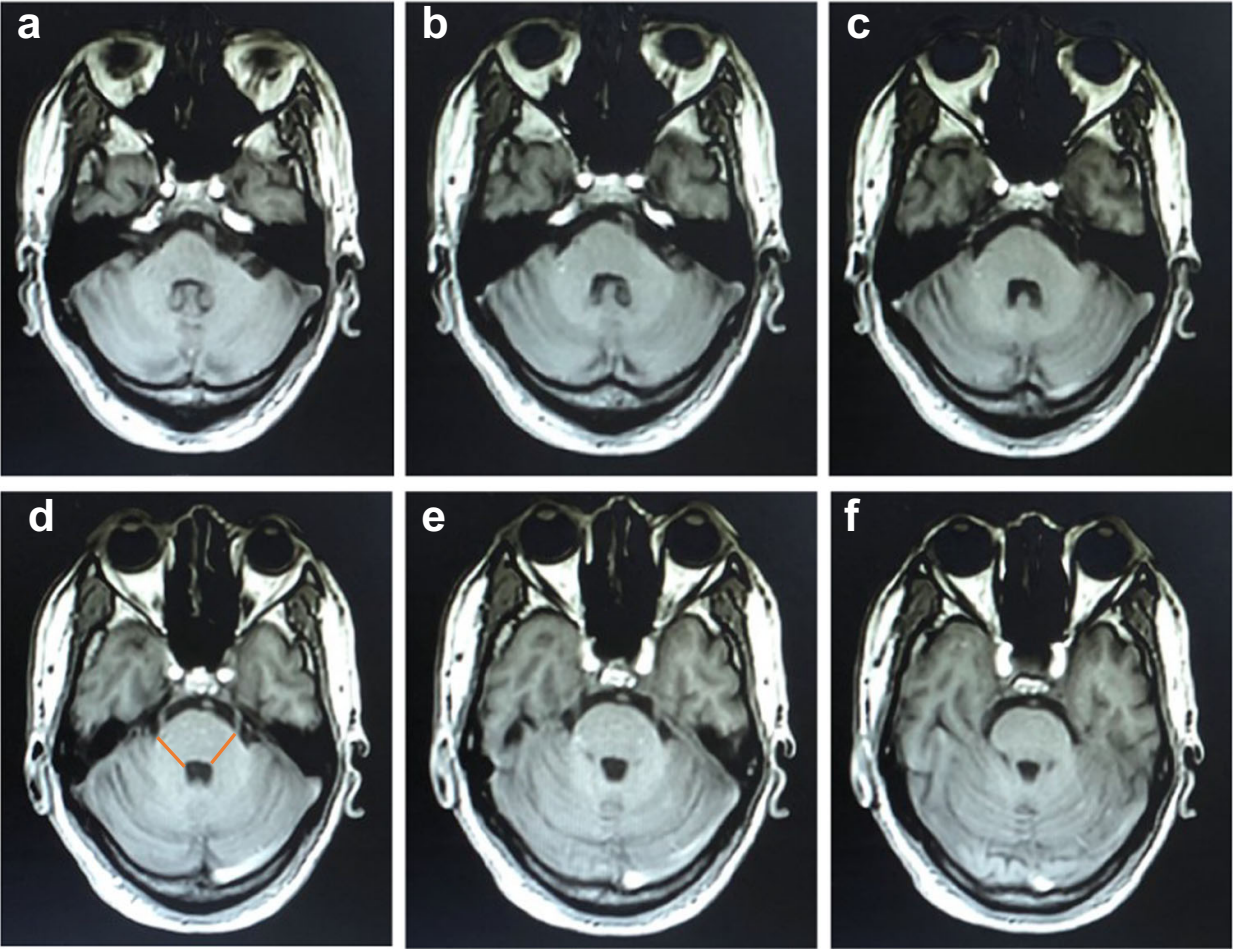
Representative three‐dimensional T1‐weighted axial images of pons and MCPs. **a**–**f** represented an axial view from the pontomedullary junction to the level of the pontine midbrain junction. Slice thickness was 2 mm, and the maximum width of MCP was shown on image **d**. The MCP value was determined by the averaged values on the widths of both MCP sides. *MCP* middle cerebellar peduncle.

**Fig. 5:**
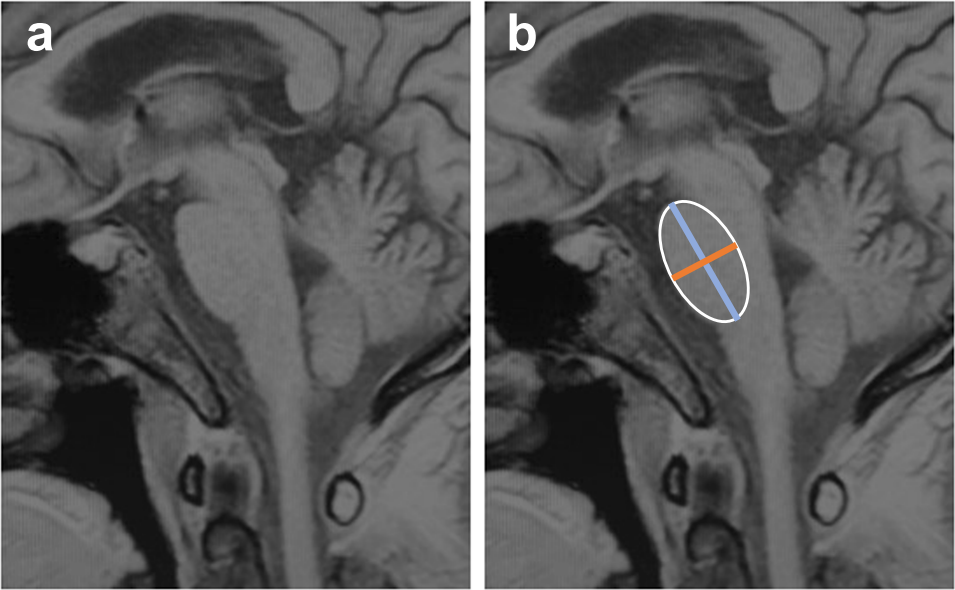
Schematic diagram of the measurement of the anteroposterior diameter of the pons. **a** T1-weighted image on conventional midsagittal MRI. **b** Regions of interest were placed over the pons and the midbrain in the midsagittal slice. Two vertical lines were drawn. Blue line defined the superior-inferior axes. Orange line represented the anteroposterior diameter.

In addition, the length of the horizontal line and vertical line of HCBs, the atrophy of pons and the atrophy of MCP were also evaluated as showed in Figs 4 and 5. Methods for measuring MCP widths was referred to Sako et al^28^. Measurements were performed using MRIcron (http://www.mccauslandcenter.sc.edu/mricro/mricron/). The slice with maximal size was adopted. Widths of both sides were averaged for the MCP. The method used for size measurements of the middle cerebellar peduncle was shown in Fig. 4. The anteroposterior diameter of pons was measured according to the method described by Massey, et al., 2013^29^. Briefly, elliptical regions were placed over the pons and the midbrain in the midsagittal slice. Two lines were drawn to define the major axes of the ellipses, corresponding to oblique superior-inferior axes (blue line, Fig. 5). The maximal measurement perpendicular to the major axis was taken, representing the anteroposterior diameter of the pons (Orange line, Fig. 5). The posterior border of the pons was clearly identifiable and did not include the pontine tegmentum. The methods used for size measurements of the anteroposterior diameter of pons are shown in Fig. 5. Two independent movement disorder specialists (Y Pan and YP Wang), who were blinded to the clinical information, evaluated the size three times, and the average value was taken. In cases of discrepancy between sizes assessed by the two Neurologists, the final size for analysis was decided by consensus between the two Neurologists. Representative brain MRI of MSA cases was shown in Figs 3–5.

### Statistical methods

We compared clinical variables between MSA-C and MSC-P group with the Mann–Whitney test or Student’s *t* test or *χ*^2^ tests when each cell had ten or more observations. We used Fisher’s exact test to compare symptoms between groups when cells had fewer than ten observations. We evaluated the correlation of HCBs with other clinical and imaging variables with spearman’s correlation analysis. Curve estimate and general linear model with univariate analysis were used to analyze the relationships between the HCBs grades and SARA scores; atrophy of pons, atrophy of MCP and disease duration were analyzed as covariates. We used multinomial ordered logistic regression to evaluate the independent risk factors for disability of MSA patients. Statistical analysis was performed with SPSS 23.0 for Windows (SPSS, Chicago, IL). The inter- and intra-rater reliability was assessed using Fleiss’ kappa and Cohen’ s Kappa analysis as implemented in the R package “irr”^30,31^.

## Supplementary information

Supplementary Table 1

## Data Availability

The data that support the findings of this study are available from the corresponding author upon reasonable request.
