# Supplementary material for: “Hot cross bun” is a potential imaging marker for the severity of cerebellar ataxia in MSA-C
Source: NPJ Parkinsons Dis. 2021 Feb 15;7:15. doi: 10.1038/s41531-021-00159-w (PMC7884406; doi:10.1038/s41531-021-00159-w)
Supplement: Supplementary file 1 — Supplementary Table 1 [file 41531_2021_159_MOESM1_ESM.pdf]

Supplementary Table 1. Inter- and intra- rater reliability of the HCBs grade evaluation

Variable	Inter-rater reliability						Intra-rater reliability				
	Rater A Grade/N/N	Rater B Grade/N/N	Rater C Grade/N/N	Z value	P-value	Fleiss' kappa	Rating 1 Grade/N/N	Rating 2 Grade/N/N	Z value	P-value	Cohen's Kappa
HCBs Grade	1 (13/81)	1 (15/81)	1 (13/81)	23	P<0.001	0.750	1 (11/90)	1 (11/90)	14.9	P<0.001	0.815
	2 (20/81)	2 (16/81)	2 (18/81)				2 (26/90)	2 (24/90)			
	3 (16/81)	3 (20/81)	3 (18/81)				3 (17/90)	3 (19/90)			
	4 (21/81)	4 (21/81)	4 (19/81)				4 (22/90)	4 (27/90)			
	5 (11/81)	5 (9 /81)	5 (13/81)				5 (14/90)	5 (9 /90)			

Inter- and intra- rater reliability of the HCBs grade evaluation. Rater A, B, C represent three radio-neurologists. Rating 1 and 2 represented that radio-neurologists evaluated an image twice.
